# The Rating of Voluntary Hospitals

**Published:** 1910-04-16

**Authors:** Stewart Johnson

**Affiliations:** Secretary.


### Institutional Letter-Box.

### THE RATING OF VOLUNTARY HOSPITALS.

*To the Editor of* The Hospital.

Sir,—I trust you will not think I am attaching too great importance to my opinions if I ask you to make a correction in the statement of views on the subject of the rating of hospitals which you attributed to me in the last number of The Hospital. You say "Mr. Johnson, of Great Ormond Street (is) decidedly against the total exemption of hospitals from local taxation." This is only half the truth. It is true that I am not in favour of the hospitals being exempted from the payment of rates at the cost of the particular borough in which they are situated, but I am in favour of the hospitals being totally exempted from the burden of local taxation at the cost of the areas from which the hospitals' patients are drawn. This, in my opinion, can best be effected by allowing the hospitals to pay rates to the local authorities and then refunding them to the hospitals from the Metropolitan Poor Fund, as was suggested at the Conference on Rating held under the auspices of the London County Council in 1907.

I am, dear sir, yours faithfully,

Stewart Johnson.

April 11. *Secretary.*

